# Plant Viruses as Adjuvants for Next-Generation Vaccines and Immunotherapy

**DOI:** 10.3390/vaccines11081372

**Published:** 2023-08-16

**Authors:** Nikolai Nikitin, Yuri Vasiliev, Angelina Kovalenko, Ekaterina Ryabchevskaya, Olga Kondakova, Ekaterina Evtushenko, Olga Karpova

**Affiliations:** 1 Department of Virology, Faculty of Biology, Lomonosov Moscow State University, Moscow 119234, Russia; 2 R-Pharm, Moscow 119421, Russia

**Keywords:** plant viruses, helical virions, icosahedral virions, structurally modified virions, adjuvants, vaccines, cancer immunotherapy

## Abstract

Vaccines are the cornerstone of infectious disease control and prevention. The outbreak of SARS-CoV-2 has confirmed the urgent need for a new approach to the design of novel vaccines. Plant viruses and their derivatives are being used increasingly for the development of new medical and biotechnological applications, and this is reflected in a number of preclinical and clinical studies. Plant viruses have a unique combination of features (biosafety, low reactogenicity, inexpensiveness and ease of production, etc.), which determine their potential. This review presents the latest data on the use of plant viruses with different types of symmetry as vaccine components and adjuvants in cancer immunotherapy. The discussion concludes that the most promising approaches might be those that use structurally modified plant viruses (spherical particles) obtained from the Tobacco mosaic virus. These particles combine high adsorption properties (as a carrier) with strong immunogenicity, as has been confirmed using various antigens in animal models. According to current research, it is evident that plant viruses have great potential for application in the development of vaccines and in cancer immunotherapy.

## 1. Introduction

Plant viruses have been widely used in biotechnology and medicine, including in vaccine design, for more than 35 years. Plant viruses are recognised by the innate immunity system and induce both cell and humoral immunity. Plant viruses are non-pathogenic for mammals and safe for human use, and no serious adverse events have been reported. Large quantities of these viruses readily accumulate in plants, and the extraction and purification of plant viruses is efficient (and economically feasible in terms of time and materials). Moreover, many plant viruses are stable, particularly under physiological conditions, so that strict adherence to the vaccine chain is not required for storage and transfer [1,2,3,4,5,6,7]. 

These unique features have, indeed, resulted in the research and development of very different applications of plant viruses, for use in both vaccine design and manufacturing; these include, but are not limited to, antigen generation (upstream) and their direct use as a vaccine or as a vaccine component (e.g., viral vectors, VLPs), and their use as agents in cancer immunotherapy. Although the focus of this review is the direct application of plant viruses as adjuvants, the first part of the review will briefly revisit other uses or provide references to the corresponding in-depth reviews.

## 2. Plant Viruses in Vaccine Manufacturing and Design

### 2.1. Plant Viruses for Antigen Generation

As mentioned above, plant viruses have been applied successfully in vaccine manufacturing. An efficient method for the rapid generation of protein in large quantities, including vaccine antigens (e.g., upstream), is transient expression using plant virus vectors. Genetic elements of plant viruses (e.g., promoters, terminators, translation enhancers and other regulatory sequences) are used widely for the construction of expression vectors. Currently, the most widely used vectors in this approach are RNA viruses, including Tobamoviruses, Potexviruses, Comoviruses and DNA-viruses, e.g., Geminiviruses [8,9,10,11,12]. For example, plant viruses, as an expression system, are used for the generation of individual proteins (antigens) for further use in subunit vaccines (e.g., PA protein of *B. anthracis*, S1 subunit protein of SARS-CoV-2 [13,14,15]).

The viral transient expression system in plants is also used to produce antigens and other reagents for diagnostic purposes in relation to various infectious diseases of humans and animals. For example, viral vector systems based on CPMV (family *Secoviridae*, genus *Comovirus*, species *Comovirus vignae*, according to the 2022 ICTV taxonomy release) have been successfully applied in the production of a stable positive control for RT-qPCR, a VLPs based on the surface proteins of CPMV containing synthetic RNA corresponding to the SARS-CoV-2 genome for COVID-19 diagnostics. Even a single laboratory-scale preparation could supply significant quantities of the reagent (positive control) for millions of runs [16].

### 2.2. Viral Particles and Chimeric Viruses

Plant viruses, as an expression system, can also be used to generate chimeric viral particles with exposed target peptides on the surface; this application was reviewed by Gasanova et al. [17] and Chen et al. [18]. Here, the chimeric virus was not only an expression vector, but also an antigen delivery system, and it possessed certain immune-modulating properties. Peptide antigens are known to interact effectively with the immune system if many copies are presented in an orderly fashion. 

Another approach utilises VLPs generated in plants using plant viruses [19]. These particles self-assemble due to viral structural proteins, even without a viral genome, and are similar to native virions in terms of their morphology and structure. Some vaccines based on plant-virus-derived VLPs have reached the clinical development stage, e.g., vaccines against rotavirus and norovirus, based on the Cowpea mosaic virus (CPMV) and Tobacco mosaic virus (TMV, family *Virgaviridae*, genus *Tobamovirus*) genome, respectively, and have successfully completed Phase I [20,21,22,23].

Of note is the fact that Medicago Inc. (Canada), which specialises in transient expression systems in plants, using a technology to generate influenza vaccines based on VLPs [24], was able to generate a VLP of the novel SARS-CoV-2 virus in the COVID-19 PHEIC context just 20 days after the sequence of the spike protein gene was received [25]. Certain genetic regulatory elements of plant viruses are used in expression cassettes in this approach [24,26].

Yet another interesting approach uses chimeric VLPs based on the capsid proteins of plant and animal viruses, which present target peptides. As such, a candidate vaccine against malaria, constructed as a chimeric VLP, containing Pfs25 protein linked with the N-terminus of an alfalfa mosaic virus (AMV, family *Bromoviridae*, genus *Alfamovirus*) surface protein and Alhydrogel (aluminium hydroxide gel) as an adjuvant, has demonstrated a good safety profile in Phase I clinical trials [27].

Finally, capsid proteins of plant viruses are also used to generate chimeric VLPs as vaccine candidates in other expression systems, i.e., not in plants but in bacteria, insect cells, etc. [28,29].

A new approach to plant virus application, which has only just begun development, is based on producing VLPs from a surface protein of Cowpea chlorotic mottle virus (CCMV, family *Bromoviridae*, genus *Bromovirus*) and heterologous RNA for transfection and translation in mammalian cells (mRNA vaccines) [30,31].

## 3. Plant Viruses as Adjuvants in Vaccines and Immunotherapy

Before delving into the focus of the review and discussing, in detail, vaccine adjuvants based on plant viruses, which are added to (i.e., mixed with) the antigen (vaccine) as an adjuvant, there should be an overview of the use of recombinant (modified) plant viruses as a platform (carrier) for covalent-bound vaccine antigens via chemical or enzyme conjugation [19].

Currently, Kentucky BioProcessing/iBio is carrying out Phase I clinical trials of two vaccines obtained via the chemical conjugation of a recombinant (modified) plant virus with an extra reaction-ready lysine (obtained by transient expression in plants) integrated into the N-terminus of the surface protein with the following vaccine antigens: CoV-RBD121-NP against COVID-19 (NCT04473690) [32] and a 4-valent influenza vaccine KBP-V001 (NCT04439695); data for the latter are yet to be published.

Since the outbreak of COVID-19, several systematic reviews of immunisation strategies have been published, and various vaccine types have been obtained using plant viruses [28,33,34,35,36,37,38]. Studies on the immunity-stimulating properties of plant virus virions, among other approaches, and their use as adjuvants for vaccines have not received similar attention. Since the application of adjuvants in vaccines (immune adjuvants) based on plant viruses that are readily available to be added to, or mixed with, the antigen (vaccine) is, as shown above, yet to be discussed, so this will be the focus of this review.

### 3.1. Helical Plant Viruses

The ability of a plant virus to act exclusively as an adjuvant, and not as a platform (e.g., VLP, vaccine constituent, etc.), has been examined for the first time on a flexible virus with a helical symmetry (Papaya mosaic virus (PapMV), family *Alfaflexiviridae*, genus *Potexvirus*). Acosta-Ramírez et al. (2008) [39] showed that the immunisation of laboratory animals via ovalbumin (OVA) or hen egg-white lysozyme (HEL), together with PapMV virions, resulted in an increase in IgG titres against the model antigens. Interestingly, in the case of HEL, the immunogenic effect, after just a single immunisation with 2 mg of protein adjuvanted with 30 µg of PapMV, was observed on day 30 after immunisation. In the case of OVA, the pronounced adjuvanted effect was observed on day 30 until day 120 after immunisation. In the current study, the adjuvant properties of PapMV were compared with the effect of 5 µg of lipopolysaccharide (LPS) and complete Freund’s adjuvant (CFA) (1:1, *v*/*v*). PapMV induced long-lasting IgG responses to HEL and to OVA. Of note is the fact that PapMV showed a more prolonged effect for HEL than LPS, but was less effective than CFA for both antigens. It is important to emphasise that the amounts of model antigens used in this study were much higher than those typically used in protein-based vaccines. PapMV in composition with OVA (as well as LPS and CFA) induced not only IgG1, but also IgG2a and IgG2b antibodies against OVA, whereas OVA alone induced only IgG1-specific antibodies. The authors have also shown that PapMV virions alone are recognised by both adaptive and innate immune response systems, and suggest that this may explain the long-lasting immune responses when used with these model antigens. IgG titres against the model antigen were detected even on day 400 of the experiment, and for the OVA adjuvanted with PapMV, the immune response to OVA was four times that shown for OVA alone. Finally, using PapMV virions as an adjuvant with a typhoid vaccine candidate (mixing of vaccine candidate with PapMV) enhanced its protective properties and the titres of all the IgG subclasses [39].

Using PapMV virions directly is not the only adjuvant approach; VLPs based on PapMV coat protein (CP) can also act as an adjuvant. As such, the addition of PapMV VLPs to both an influenza virus vaccine candidate [40] and a commercial influenza trivalent inactivated vaccine (TIV) [41] increased the immune response to the influenza virus. Of note is the fact that the PapMV VLPs discussed above are expressed and assembled in *E. coli* and contain RNA of bacterial origin [42]. Most importantly, in addition to the adjuvant effect demonstrated in the studies, these PapMV VLPs are also characterised by a lack of local adverse reactions compared with the aluminium-based adjuvant used in laboratory practice (Imject Alum) [40].

In recent studies, slightly different PapMV VLPs based on PapMV CP and synthetic RNA have been used successfully. This type of PapMV VLP is assembled in vitro and consists of PapMV CP expressed in *E. coli* and the non-coding ssRNA in vitro transcript [43,44,45,46]. Mathieu et al. (2013) [43] demonstrated that the administration of PapMV VLPs to mice using the intranasal route triggered a strong innate immune response. The analysis of bronchoalveolar lavage showed the secretion of TNF-α, IL-6 and IL-9, as well as different chemokines (MIP-1α, MIP-1β, MIP-2 and IP-10) and the recruitment of neutrophils, monocytes/macrophages and lymphocytes. In this study, the authors also demonstrated that animals receiving intranasal PapMV VLPs could survive an influenza and Streptococcus pneumoniae challenge. It is important to note that the monomer forms of PapMV CP or ssRNA alone were unable to induce the same effect [43]. It was also shown that PapMV VLPs with synthetic ssRNA stimulated an innate immune response through toll-like receptors (TLR) 7/8 [47]. Interestingly, Carignan et al. (2015) [48] demonstrated that the addition of this type of PapMV VLP to the PapMV-sM2eVLPs (a vaccine candidate against the influenza virus) did not enhance the immune response to the M2e, which contradicted previous results [40]. Such differences may have been due to the different dosages used in the studies, as well as to slight differences in the PapMV VLPs and PapMV-M2e VLPs used [40,48]. In another study, Bolduc et al. (2018) [44] demonstrated that the immunisation of BALB/c mice with nanoparticles from the NP protein of the influenza virus and using PapMV VLPs (with synthetic ssRNA) as an adjuvant component led to good protective efficacy against 6 and 12 LD50 of BALB/c mouse-adapted influenza A/WSN/33 (H1N1) [44]. PapMV VLPs can be used not only in vaccine design, but also in cancer immunotherapy. Lebel et al. (2016) [49] demonstrated that the administration of PapMV VLPs potentiated an anti-tumour immune response, significantly slowed melanoma progression and prolonged the survival of mice. Furthermore, the systemic administration of PapMV VLPs reduced metastasis implantation in mice lungs [49]. It is worth mentioning that the rights to exploit PapMV technology in vaccines and anti-tumour treatment have been obtained by the start-up company FOLIA BIOTECH INC., which was founded by the inventor of this technology, Dr. Denis Leclerc (WO2012155261, WO2012155262A1, EP-3046565-A4, CA-2924819-A1, CA2669485A1, WO2008058396A1). The safety of PapMV VLPs using synthetic ssRNA for humans was shown in a Phase I trial (NCT02188810) [45]. Technology based on PapMV is very promising, but it is worth emphasising that most of the relevant research has been carried out on PapMV VLPs, rather than PapMV virions. The size of PapMV VLPs, whose average length is generally up to ~150 nm, and PapMV, whose length is 530 nm, differs considerably [40,42,43,50]. It is certainly safest, for the environment, to use PapMV VLPs based on non-coding RNA, but it would nevertheless be interesting to carry out a comparative study of the adjuvant properties of PapMV VLPs, PapMV virions and UV-treated PapMV virions. Acosta-Ramírez et al. (2008) [39] demonstrated that both PapMV virions and UV-inactivated PapMV induced a strong T-cell response (delayed-type hypersensitivity response), but it would be interesting to see a wider study that also included PapMV VLPs [39].

Other interesting results have been reported for plant viruses of the *Potyviridae* family. The possibility of using potyviruses as an adjuvant was demonstrated for the first time on the Tobacco etch virus (TEV, family *Potyviridae*, genus *Potyvirus*). In this study, it was shown that TEV activated T-cell and IgG responses when administered alone via the intraperitoneal route to mice. The highest titres, after the immunisation of laboratory animals, were for the IgG2a isotype. In an experiment using cultured splenocytes, it was shown that TEV induced interferon γ (IFNγ) secretion [51]. This fact, in combination with high IgG2a titres, enables TEV to be regarded as an adjuvant capable of stimulating a Th1 response. In another study, the authors analysed the adjuvant properties of TEV VLPs produced in *E.coli*. These VLPs were based on TEV CP and contained *E.coli* RNA; their length varied from 20 nm to 2 μm (mean 553 nm). It was shown that TEV VLPs can modulate the immune response and change the IgG2/IgG1 ratio against the antigen (Porcine respiratory and reproductive syndrome virus (PRRSV, family *Arteriviridae*, genus *Arterivirus*) chimeric protein) towards IgG2a, in comparison with the antigen alone. Interestingly, the isotype with the highest titre was IgG1, which differed from the results obtained previously for TEV virions. This may have been due to a different route of administration (subcutaneous in the case of TEV VLPs), differences between TEV virions and TEV VLPs, or the nature of the PRRSV chimeric protein with which the animals were immunised in composition with the TEV VLPs [52]. VLPs based on the CP of another potyvirus, Papaya ringspot virus (PRSV, family *Potyviridae*, genus *Potyvirus*), have also been demonstrated to have adjuvant potential. The immune response to the mixture of PRSV VLPs using a peptide derived from the influenza virus hemagglutinin (HA) has been shown to be comparable with the human trivalent influenza vaccine (FLUZONE 2013–2014, Sanofi Pasteur). The immune response against the HA peptide after immunisation with PRSV VLPs and the peptide mixture was significantly higher than in the case of immunisation using the peptide alone [53].

The adjuvant properties of two other viruses with virions of helical symmetry were examined using model antigens (OVA and/or HEL). These were the rod-like Tobacco mosaic virus and the filamentous Potato virus X (PVX, family *Alphaflexiviridae*, genus *Potexvirus*). It was demonstrated that TMV possessed adjuvant properties and increased total IgG titres against OVA in comparison with individual OVA. HEL and OVA were used in analyses of the immunostimulating properties of PVX, and its virions did not show adjuvant potential in composition with these antigens [54]. The possibility of immunostimulating an anti-tumour response against melanoma using TMV was also shown [55].

Information about the immune response to the plant-virus-based adjuvant used may be necessary for the design of vaccine formulations, but an assessment of the ratio of antibody titres to the antigen and adjuvant is not often reported in publications. Among the helical plant viruses and their VLPs that have been studied exclusively as adjuvants, data are available for TMV, PVX and TEV VLPs [52,54]. It has been shown that relatively high titres of IgG are produced on PVX with no significant enhancement of the immune response to the model antigen. In contrast, no significant immune response was detected on TMV, while there was an effective increase in the immune response to the model antigen [54]. In the case of TEV VLPs, antibody titres to the adjuvant and antigen were comparable [52]. It is assumed that having fewer antibodies induced to the adjuvant makes the immune response to the vaccine antigen more favourable.

### 3.2. Structurally Modified Helical Plant Viruses 

A promising adjuvant in the development of recombinant vaccines is represented by structurally modified plant viruses—spherical particles (SPs)—formed during the thermal remodelling of the Tobacco mosaic virus (TMV) [54,55,56,57,58]. The safety of the intravenous, intramuscular and intraperitoneal administration of SPs has been shown in various studies [2,6,7]. Plant viruses and their structurally modified particles are assumed to manifest immunostimulating properties because they consist of proteins antigenically alien to the mammalian immune system. Nevertheless, their regular structure, formed by identical subunits, could be even more important [59,60,61]. For example, a comparison of the influence of the intratumoural administration of TMV, SPs and TMV CP (subunit) on tumour development has shown that TMV and SPs can delay tumour growth, with immunisation using TMV CP having no effect. The authors of the study suggest that it is the multivalent nature of plant virions and plant virus-like particles, regardless of their shape and size, that plays a key role in activating anti-tumour innate immunity, by stimulating pattern recognition receptors [61]. Thus, it is likely that the regular, repeated structure that is inherent in plant viruses and in their structurally modified particles is key to their interaction with pattern recognition receptors. Because of this, they can intensify the uptake of surrounding antigens using antigen-presenting cells that lead to further humoral and cellular immune response activation.

Antigen adsorption on the SPs’ surface might also influence their ability to serve as an adjuvant. A number of published studies have reported individual proteins, with various charges and molecular weights, being absorbed onto the SPs’ surfaces [54,55,56,62,63,64], as well as the possibility of the simultaneous adsorption of two [55,65], or even more [58], recombinant proteins. Without additional fixation, adsorbed antigens can dissociate from the SPs’ surface; this could, for example, be mediated by changes to the solution’s pH or ionic strength. Formaldehyde treatment has been shown to lead to the formation of an SPs–antigen complex with covalent bounds between the antigen and SPs’ surface [55]. The immunostimulation activity of SPs has been detected within both SPs + antigen(s) compositions and fixed SP–antigen complexes [54,55,56,57,58]. Vaccine composition, the production of which does not require the additional stage of formaldehyde treatment, seems to be more reasonable. Moreover, it is assumed that, in this case, antigens can gradually dissociate from the surface of the SPs. Thus, SPs can likely act as an antigen depot and play an additional positive role in stimulating the immune response.

In the context of model antigens (ovalbumin, Potato virus X coat protein) and the recombinant antigens of various viruses causing human infectious diseases (rubella, COVID-19), SPs have been shown to effectively stimulate the immune response to the target antigens administered using them. Their ability to induce SARS-CoV-2-neutralising antibodies (NAb) was shown for a betacoronavirus vaccine candidate [58]. Moreover, SPs are able to enhance the immune response to inactivated viruses (Puumala virus, Rabies virus) [66,67]. Adding SPs to the inactivated Puumala vaccine formulation was revealed to significantly enhance the induction of virus-specific NAb [66]. In a study by Nikitin et al. (2018c) [67], the ability of SPs to enhance the protective efficacy of the veterinary Rabies inactivated vaccine “Rabican” was demonstrated *in vivo*. The adjuvant effect of SPs was comparable with the incomplete Freund’s adjuvant that was used as a positive control [67]. In a recent study by Granovskiy et al. (2022) [68], for the first time, the protective efficacy of an SP-based recombinant vaccine candidate was proven in in vivo studies. The SP-based anthrax vaccine candidate was shown to provide up to 100% protection in guinea pigs against the spore challenge of the fully virulent *Bacillus anthracis* strain 81/1 (pXO1+, pXO2+) [68]. 

The preexisting antibodies specific to the excipient vaccine components (adjuvant/platform/vector), which were elicited via the first immunisation, can decrease the efficacy of boost immunizations with the same vaccine formulation and the efficacy of the following vaccinations with formulations based on the same technology. In this regard, it is important to evaluate the magnitude of the immune response to the adjuvant/platform. In a number of experiments, the ratio of antibody titres to antigens, which were administrated using SPs, and to SPs was found to be from 6:1 to 150:1 [54,57,58] (Table 1). The relatively low titres to SPs ratio gives hope that SPs may be used in vaccines intended to boost immunisations, and in more than one vaccine included in the National Vaccination Schedule.

A number of studies have provided evidence that SPs can stimulate both humoral and cellular branches of the immune response [57,58,66]. The effective stimulation of the production of immunoglobulins by the various antigens administered in compositions with SPs indicates the positive activation of humoral immunity [54,57,58,66]. At the same time, the injection of SPs in the absence of any antigen was shown to lead to the enhancement of IL-1β, INFγ and IL-12-level production in mice [66]. IL-12 is a crucial driver of CD4+ T lymphocytes differentiation to Th-1 cells, while IL-1β is a potent proinflammatory cytokine. INFγ is a Th1 cytokine, mediating the elimination of virus-infected cells. Thus, the raising of these cytokine concentrations indicates Th-1 response activation. Vaccination with SPs + inactivated Puumala virus compositions resulted in a statistically significant higher production of IL-1β and IL-12 in comparison with vaccination using inactivated Puumala virus without adjuvant. The addition of SPs into the vaccine candidate formulation did not influence INFγ induction [66]. It is interesting to note, however, that aluminum hydroxide, which is considered to provide a Th2-biased immune response [69], significantly reduced INFγ and IL-12 production in comparison with inactivated Puumala virus without adjuvant [66].

An evaluation of the IgG1/IgG2a ratio in mouse model experiments has provided circumstantial evidence as to which branch of the immune response has been predominantly activated. The presence of various IgG subclasses was revealed in mice sera after immunisation with SP + recombinant Rubella virus antigen (tetraepitope A—A4) compositions. However, higher titres of IgG1 in comparison with those of IgG2a enabled the authors to suggest that SPs were more likely to stimulate a Th2-biased immune response [57]. Similarly, in a study by Evtushenko et al. (2020) [54], the majority of OVA-specific IgG, which were induced via immunisation with SPs + OVA compositions, belonged to the IgG1 subclass. After immunisations with the betacoronavirus vaccine candidate (SPs + three coronavirus antigens), all subclasses of IgG (IgG1, IgG2a, IgG2b and IgG3) specific to the each of the coronavirus antigens were detected. Nevertheless, a considerable prevalence of IgG1 or IgG2a was not observed, and the authors supposed that SPs induced the well-balanced Th1/Th2 response to coronavirus antigens [58]. Summarising the data obtained from various research studies, it can be concluded that SPs are probably able to activate both a humoral and a cellular immune response.

Studies describing the adjuvant efficacy of SPs in relation to a wide range of antigens that differ in nature and size are summarised in Table 1. Various doses of SPs, means of administration and immunisation schedules were applied. Immunostimulatory potential was demonstrated for SPs with a diameter ranging from 100 nm to 611 nm. SPs of a smaller or larger size could also possess immunostimulation properties. Although the issue was not specifically addressed, it is most likely that existing data show that size has no impact on SPs’ adjuvant potential. 

Conversely, it is likely that the SPs dose, as well as the SPs and antigen mass ratio, influence the effectiveness of immunostimulation. For example, the adjuvant effect of SPs on inactivated Puumala virus was only clear when the SPs dose was 150 μg per animal. In this case, the total protein of the Puumala virus (3.455×10^4^ inactivated virus particles) was 19 μg; therefore, the SPs/antigen protein mass ratio was 7.9:1. The quantity of 75 μg of SPs added to the same amount of inactivated virus (protein mass ratio—3.9:1) was shown to have a significantly weaker adjuvant effect. The addition of 50 μg of SPs per dose (protein mass ratio—2.6:1) had no positive impact on NAb production, nor did it decrease the effectiveness of antibody production when compared with inactivated Puumala virus without adjuvant. The stimulation of the release of cytokines was detected only at 150 μg of SPs per dose [66]. 

In another study, the addition of 100 μg of SPs to the inactivated Rabies vaccine “Rabican” (1.8 IU) enhanced its protective efficacy [67]. The same amount of SPs (100 μg) was used for intratumoural vaccination, which resulted in the decay of tumour growth and an increase in survival time [61]. Furthermore, 100 μg of SPs per dose was shown to be effective for the enhancement of the humoral immune response to the Rubella recombinant antigen (A4) [57] and ovalbumin (OVA) [54]. The titres of antigen-specific total IgG were 10 and 15 times higher in the case of immunisations using SP-containing compositions than those using individual A4 and OVA, respectively. The SPs/antigen mass ratio was 10:1 for A4 (10 μg of A4 per dose) and 20:1 for OVA (5 μg of OVA per dose) [54,57]. 

In a recent study by Kovalenko et al. (2022) [58], a close SPs/antigen mass ratio (11.9:1) was used for the generation of a coronavirus vaccine candidate. One dose included 250 μg of SPs and 21 μg of three betacoronavirus recombinant antigens (7 μg each) [58]. The same dose of SPs (250 μg) was revealed to be effective for improving antibody production in response to Cauliflower mosaic virus (CaMV, family *Caulimaviridae*, genus *Caulimovirus*). Despite CaMV being highly immunogenic on its own, the immune response to the virions was higher when immunisation was carried out with CaMV being used in combination with SPs (CaMV—10 μg, SPs/CaMV mass ratio—25:1) than when immunisation occurred using the same amount of individual CaMV [56].

A vaccine candidate containing 300 μg of SPs and 30 μg of *Bacillus anthracis* recombinant protective antigen (rPA83m) (SPs/antigen mass ratio—10:1) per dose protected guinea pigs from a fully virulent *B. anthracis* strain challenge. In comparison with individual rPA83m, there was no significant SPs contribution to the vaccine candidate’s immunogenicity and protectiveness. The authors suggest, however, that this result could be explained by the excessive content of rPA83m per dose, which was sufficient to reach the upper limit of the immune response, even without adjuvant. It is noteworthy that, in the same study, SPs were shown to have a significant stabilising effect on rPA83m. Furthermore, SPs were observed to reduce a range of rPA83m-specific antibody titre values after immunisation with a stored vaccine candidate [68]. 

A dose of 500 μg of SPs in combination with 20 μg of Potato virus X coat protein (PVX CP) (SPs/PVX CP mass ratio—25:1) produced an adjuvant effect related to PVX CP. A similar result was observed for recombinant PVX CP fused with the plum pox virus (PPV) epitope [55].

In summary, 100–500 μg of SPs per dose and SPs/antigen mass ratios ranging from 7:1 to 25:1 could be considered to be appropriate to provide an effective immune response to the antigen (Table 1). Nevertheless, the selection of the most effective SPs dose and SP/antigen ratio in a vaccine formulation requires careful attention and is, perhaps, dependent on antigen features.

### 3.3. Icosahedral Plant Viruses

In addition to plant viruses with a helical structure, a number of studies have evaluated the potential of icosahedral plant viruses, both as adjuvants in vaccines and for cancer immunotherapy. Evtushenko et al. (2020) [54] examined the adjuvant potential of two icosahedral viruses, CaMV and Bean mild mosaic virus (BMMV, family *Tombusviridae*). CaMV and BMMV have icosahedral virions with a similar diameter, of about 35 nm, but a different type of viral genome (DNA and RNA for CaMV and BMMV, respectively) [70]. Unlike BMMV, CaMV, in a mixture with a model antigen, demonstrated the induction of a strong immune response. The authors suggest that this difference was probably related to the type of genomic nucleic acid (DNA or RNA). At the same time, both viruses induced a high self-immune response. Although in the case of CaMV this property does not prevent the stimulation of the immune response to model antigens, there is a need for additional analyses of the potential systemic toxicity risks of viral particles in order to assess their applicability in the biomedical context. In the same study, the authors conducted a comparative analysis of the adjuvant potential of icosahedral viruses with helical plant viruses (TMV and PVX) and found no significant difference in their immunostimulating properties using model antigens, concluding that the virion shape had no effect on adjuvant properties [54].

The immunostimulating effect of the Cowpea mosaic virus and the possibility of its use as a cancer vaccine and in immunotherapy are actively being studied by a group led by Dr. N.F. Steinmetz. CPMV has an icosahedral capsid that is 30 nm in diameter and an RNA genome. The authors have demonstrated the effectiveness of CPMV virions and VLPs in cancer immunotherapy in syngeneic mice tumour models of melanoma and glioma, and breast, ovarian and colon cancer [61,71,72,73,74,75,76,77,78], as well as in companion dogs with oral melanoma [79]. The introduction of CPMV contributed to a significant delay in, or prevention of, tumour progression, and led to increased survival rates in laboratory animals. A study of the mechanism of CPMV immunostimulation activity indicated that the virus initiates the activation of innate anti-tumour immunity via MyD88-dependent TLRs 2, 4 (which recognise the viral capsid) and 7 (which recognises the viral ssRNA). Depending on the model studied, the mechanism of action is based on various combinations of neutrophils, antigen-presenting cells, adaptive immunity cells, interleukin IL-12, type I interferons and/or interferon (IFN)-γ [71,74,80,81,82].

The effectiveness of the antitumour effect of native CPMV, inactivated virions (chemically or with ultraviolet light) and VLPs assembled from capsid proteins in the absence of genomic RNA was compared. Despite the maintenance of a high level of immunostimulation activity in all variants, native CPMV virions were the most effective, which indicates that viral RNA provides additional signalling through TLR-7/8, which increases the effectiveness of this adjuvant [83,84,85].

Several studies have used a combined immunotherapeutic approach to induce a higher antitumour immune response. Patel et al. (2018) [86] applied an approach that combined the intratumoural injection of CPMV with radiation therapy on a syngeneic mouse model of ovarian carcinoma. Such a combination therapy resulted in a more effective delay in tumour growth, compared with individual exposure to CPMV or radiation therapy [86]. The same group of scientists demonstrated the synergistic effect of combined therapy using CPMV and anti-PD-1 antibodies, or agonistic OX40-specific antibodies (checkpoint inhibitors), using three tumour models (i.e., mouse models of ovarian cancer, colon cancer and melanoma) [77]. In another study, the authors showed the effectiveness of a combination of CPMV immunisation and chemotherapy in the context of low doses of cyclophosphamide as a combination therapy for breast tumours in mice in vivo [72]. It has also been shown that the combination of CPMV and the irradiation of mouse cancer cells is an effective approach to prevention in an aggressive murine ovarian cancer model. The effectiveness of such a combination in this study surpassed other combinations of CPMV using lysates of tumour cells prepared in different ways (freeze–thawed lysates, heat-shocked lysates and HOCl-oxidised lysates) [78]. Thus, the combination of conventional approaches with the immunostimulating properties of CPMV could contribute to the more effective immunotherapy of various types of cancer.

In order to prolong the immunostimulating effect of the virus in the organism, as well as to prevent rapid viral degradation and the need for repeated injections, a method of CPMV formulation in a hydrogel based on chitosan and glycerophosphate was proposed. Such a composition enabled the local retention of CPMV in the intraperitoneal space and the release of the virus from the hydrogel three weeks after administration (in contrast, using CPMV alone enabled the release of the virus one week after administration). In a colon cancer mouse model, the combination of alone and hydrogel-formulated CPMV has been shown to be effective in preventing tumour growth [87,88].

Studies of the adjuvant activity of CPMV and analyses of its biodistribution, toxicology and clearance in mice have demonstrated the safety of CPMV for biomedical applications [89,90].

Immunostimulating properties have also been studied for the alfalfa mosaic virus. AMV virions consist of a mixture of bacilliform and spherical particles containing individual genomic RNA segments. The absence of AMV cytotoxicity in vitro and its high efficacy as an immunostimulator and inducer of the anti-tumour immune response in situ were shown in a mouse model of breast cancer [91].

Beiss et al. (2022) [92] compared the immunostimulatory effect of CPMV with other plant viruses from the same *Secoviridae* family—Cowpea severe mosaic virus (CPSMV, genus *Comovirus*, species *Comovirus severum*) and Tobacco ringpot virus (TRSV, genus *Nepovirus*, species *Nepovirus lycopersici*, according to the 2022 ICTV taxonomy release) [92]. CPMV was significantly more effective than CPSMV and TRSV in a mouse model of dermal melanoma, which was probably due to CPMV signalling through TLR-7 (and hence IFNβ), in contrast to CPSMV and TRSV, which predominantly signal through TLR-2 and TLR-4. In addition, CPMV induced a higher level of, and a more prolonged, intratumoural induction of cytokines than other plant viruses. Another study compared the immunogenicity of three icosahedral plant viruses with similar virion sizes (about 30 nm) and belonging to different families—CPMV, CCMV and Sesbania mosaic virus (SeMV, family *Solemoviridae*, genus *Sobemovirus*). Immunostimulatory properties have been demonstrated for all three viruses. Unlike CCMV and SeMV, only CPMV induced the secretion of all cytokines tested in the study. This may indicate the greater efficacy of CPMV when used as an adjuvant [93]. The same set of plant virus virions and the VLP of the Physalis mottle virus (PhMV, family *Tymoviridae*, genus *Tymovirus*, species *Tymovirus physalis*) were used to evaluate the effectiveness of viral particles as in situ immunostimulants in tumour models of melanoma, ovarian cancer and colon cancer. Similarly, the greatest efficacy in delaying tumour growth occurred after the administration of CPMV, while CCMV and SeMV showed more modest results. The efficacy of PhMV VLP was comparable in its effect to the PBS group [74]. Thus, all the presented results indicate the immunostimulatory effect of a number of icosahedral viruses and their effects on tumour development. However, CPMV clearly stands out as having demonstrated a high level of efficacy and having great potential in the development of biomedical applications in the field of antitumour therapy.

When plant viruses are used as cancer immunotherapy agents, there is a risk that their immunostimulatory efficacy will be reduced by the presence of antibodies obtained using food or via repeated virus administration. In contrast, antibodies that pre-existed when CPMV was administered appeared to increase its efficacy (as indicated by animal survival) in a syngeneic mouse model of ovarian cancer in situ [94]. Similar results were demonstrated for CCMV VLPs that included self-amplifying (“replicon”) mRNA [31]. Apparently, plant-virus-based formulations can also be used in cancer immunotherapy in a prime-boost immunisation strategy.

## 4. Conclusions

Published studies enable an unambiguous conclusion to be made, indicating that plant viruses are of great interest for biotechnology and biomedicine, particularly for vaccine development and cancer immunotherapy (Table 2). A considerable amount of accumulated data show that SPs, VLP and virions (belonging to various taxonomy groups) produce an adjuvant effect in relation to a wide range of antigens that differ in nature and size. Moreover, a covalent bounding of antigen (antigens) with the SPs’ surface does not require the effective stimulation of the immune response. Moreover, plant viruses of different shapes (symmetry) and sizes have been demonstrated to have great potential for inducing both local and systemic anti-tumour immune responses. This occurs by overcoming the suppression of immunity, which restricts the immune system’s ability to eliminate pathologic cells. The anti-tumour activity of various plant viruses has been demonstrated using in vivo and in vitro experimental models, while plant viruses have been shown to be more effective than their VLP. Unfortunately, the application of plant virus virions for vaccine design and for cancer immunotherapy has not yet been implemented to an appropriate extent. There is some apprehension related to the application of native infection virions. In this regard, however, it should be stated that there is clear evidence of the complete safety of plant viruses. The introduction of standardised protocols for chemical or UV RNA genome inactivation could facilitate the promotion of preventive and therapeutic medications based on plant virus native virions.

## Figures and Tables

**Table 1:** The designs of studies that have shown plant virus structurally modified particles’ adjuvant efficacy.

Amount of SPs (SPs Size)	Antigen Description	SPs/Antigen Mass Ratio (Calculated Based on Protein Content)	Dose Volume (Administration Solution)	Animal Model, Route of Administration and Number of Immunisations	Impact on Immunogenicity and/or Protectiveness in Comparison with Individual Antigen	Reference
150 μg (260 nm)	Inactivated Puumala virus—19 μg (3.455 × 10^4^ particles of inactivated virus)	7.9:1	0.5 mL (PBS)	Mice, intramuscularly, 2 immunisations with 14 days interval	Enchantment of NAb induction, enchantment of IL-1β and IL-12 induction	[66]
75 μg (260 nm)		3.9:1			Weak enchantment of NAb induction	
50 μg (260 nm)		2.6:1			Negative impact on immunogenicity	
100 μg (285 nm)	Inactivated Rabies vaccine «Rabican» (1.8 IU)		0.5 mL (Hank’s medium)	Mice, intraperitoneal, two immunisations with seven days interval	Increase protective efficacy	[67]
100 μg (250 nm)	-	n/a	0.02 mL (PBS)	Mice, eight days post tumour induction mice were treated intratumourally every 4 days	Slow tumour growth and increase survival time	[61]
100 μg (299 nm)	Rubella virus recombinant antigen A_4_ (22 kDa)—10 μg	10:1	0.1 mL (PBS)	Mice, intramuscularly, three immunisations with 14 days interval	Enhancement of immune response to A_4_ (A_4_/SPs total IgG ratio: 6/1)	[57]
100 μg (611 nm)	Ovalbumin (OVA) (42.7 kDa)—5 μg	20:1	0.2 mL (PBS)	Mice, subcutaneously, four immunisations with 14 days interval	Enhancement of immune response to OVA (OVA/SPs total IgG ratio: 47/1)	[54]
250 μg (466 nm)	Coronavirus recombinant antigens (three antigens—21 μg total): Co1 (27.5 kDa)—7 μg, CoF (30.5 kDa)—7 μg, PE (18.8 kDa)—7 μg	11.9:1	0.2 mL (PBS)	Mice, intraperitoneal, two immunisations with 14 days interval	Enhancement of immune response to CoF and PE (CoF/SPs total IgG ratio: 9/1; PE/SPs total IgG ratio: 16/1)	[58]
250 μg (308 nm)	CaMV—10 μg	25:1	0.5 mL (H_2_O)	Mice, intraperitoneal, three immunisations with 14 days interval	Enhancement of immune response to CaMV	[56]
300 μg (435 nm)	Anthrax recombinant protective antigen rPA83m (84 kDa)—30 μg	10:1	0.3 mL (PBS)	Guinea pigs, subcutaneously, two immunisations with 14 days interval	Reduction of specific to rPA83m antibody titres values range after immunisations with stored vaccine candidate (rPA83m/SPs total IgG ratio: from 94/1 to 150/1)	[68]
500 μg (100 nm and 500 nm)	PVX CP or PVX recombinant CP fused with PPV epitope—20 μg	25:1	0.4 mL (H_2_O)	Mice, intraperitoneal, two immunisations with 14 days interval	Enhancement of immune response to PVX CP or PVX recombinant CP fused with PPV epitope	[55]

Nab—neutralizing antibodies, CaMV—Cauliflower mosaic virus, PVX—Potato virus X, PPV—Plum pox virus, CP—coat protein.

**Table 2:** Studies of plant viruses as tools for vaccine development and cancer immunotherapy.

Plant Virus Virions	Antigen/Disease	Highlights	References
PapMV (*Potexvirus*)	OmpC of *S. typhi*; model antigens (ovalbumin, OVA/hen egg lysozyme, HEL)	Protectivity increasing against *S. typhi*, enhancement of antibody response to model antigens, characterisation of immune response to PapMV.	[39]
PapMV VLPs with *E.coli* RNA (*Potexvirus*)	Influenza virus vaccine candidate; commercial influenza TIV	Enhancement of immune response to influenza virus.	[40,41]
PapMV VLPs with non-coding synthetic RNA (*Potexvirus*)	Influenza; *Streptococcus pneumoniae*	Triggering of strong innate immune response; protectivity in *S. pneumoniae* and influenza challenges.	[43,44]
	*Listeria monocytogenes*	Immune activation through TLR7; increasing of protection against a *Listeria monocytogenes.*	[47]
	No injected antigen/melanoma	Potentiation of anti-tumour immune response; PapMV VLPs significantly slowed down melanoma progression and prolonged survival of mice.	[49]
	Commercial influenza TIV	Phase I clinical trials to assess the safety of PapMV VLPs combined with TIV (NCT02188810).	[45]
TMV (*Tobamovirus*)	Model antigen (OVA)	Enhancement of immune response to model antigen. Comparison of the adjuvant properties of TMV with other plant viruses.	[54]
	No injected antigen/melanoma	TMV inhibited tumour growth and increased survival time. The effectiveness of TMV compared to CPMV was significantly lower.	[61]
TEV (*Potyvirus*)	No antigen	Characterisation of immune response to TEV.	[51]
TEV VLPs with *E.coli* RNA (*Potyvirus*)	PRRSV chimeric protein	Modulation of the immune response and changing of the IgG2/IgG1 ratio against antigen.	[52]
PRSV (*Potyvirus*)	Influenza virus HA peptide	Enhancement of immune response to antigen.	[53]
PVX (*Potexvirus*)	Model antigens (OVA, HEL)	No enhancement of immune response to model antigen. Comparison of the adjuvant properties of PVX with other plant viruses.	[54]
AMV *(Alfamovirus)*	No injected antigen/breast cancer	Evaluation of anti-cancer efficacy. AMV activates multiple innate immune responses and anti-tumour T-cell responses.	[91]
CPMV (*Comovirus*)	No injected antigen/melanoma	CPMV elicits potent anti-tumour immunity. Comparison of the immunostimulatory properties of CPMV with TMV.	[61]
	No injected antigen/melanoma, breast cancer, ovarian cancer, intracranial glioma, colon adenocarcinoma, colorectal carcinoma	CPMV itself induces significant systemic anti-tumour immune-mediated efficacy. Comparison of the immunostimulatory properties of wild-type CPMV and eCPMV (RNA-free VLPs).	[71,73,74,75,76,81,82]
	Irradiated tumour cells/ovarian cancer	Co-delivery of CPMV with irradiated ovarian cancer cells constitutes an effective prophylactic anti-tumour vaccine. Comparison of the adjuvant properties of CPMV in combination with freeze–thawed lysates, heat-shocked lysates, and HOCl-oxidised lysates of tumour cells.	[78]
	No injected antigen/ovarian cancer	Combination of radiation therapy with CPMV demonstrates the potent anti-tumour efficacy.	[86]
	No injected antigen/breast cancer	Combination of CPMV with cyclophosphamide (CPA) chemotherapy demonstrates synergistic anti-tumour efficacy.	[72]
	Checkpoint-targeting antibodies/ovarian cancer, colon cancer, and melanoma	Combination of CPMV with a PD-1 inhibitor or OX40 agonist showed greater therapeutic anti-tumour efficacy than monotherapy.	[77]
	No injected antigen/colon cancer	Evaluation of the anti-tumour preventive efficacy of CPMV in injectable hydrogels (chitosan and glycerophosphate).	[87,88]
	No injected antigen/melanoma	Study on the efficacy and mechanism of action of CPMV compared to inactivated CPMV treated with UV light, β-propiolactone or formalin.	[83,84,85]
	No injected antigen/ovarian cancer	Anti-tumour efficacy of CPMV was enhanced in the presence of pre-existing antibodies.	[94]
CCMV (*Bromovirus*)	No injected antigen/melanoma, ovarian cancer and colon cancer	Evaluation of anti-tumour efficacy and comparison with CPMV.	[74]
SeMV (*Sobemovirus*)	No injected antigen/melanoma, ovarian cancer and colon cancer	Evaluation of anti-tumour efficacy and comparison with CPMV.	[74]
CPSMV (*Comovirus*)	No injected antigen/melanoma	Evaluation of anti-tumour efficacy and comparison with CPMV.	[92]
TRSV (*Nepovirus*)	No injected antigen/melanoma	Evaluation of anti-tumour efficacy and comparison with CPMV.	[92]
CaMV (*Caulimovirus*)	Model antigens (OVA, HEL)	Enhancement of immune response to model antigen. Comparison of the adjuvant properties of CaMV with other plant viruses.	[54]
BMMV (*Tombusviridae*)	Model antigens (HEL)	No enhancement of immune response to model antigen. Comparison of the adjuvant properties of BMMV with other plant viruses.	[54]

Abbreviations: PapMV (Papaya mosaic virus), TMV (Tobacco mosaic virus), TEV (Tobacco etch virus), PRRSV (Porcine respiratory and reproductive syndrome virus), PRSV (Papaya ringspot virus), PVX (Potato virus X), AMV (Alfalfa mosaic virus), CPMV (Cowpea mosaic virus), CCMV (Cowpea chlorotic mottle virus), SeMV (Sesbania mosaic virus), CPSMV (Cowpea severe mosaic virus), TRSV (Tobacco ring spot virus), CaMV (Cauliflower mosaic virus), BMMV (Bean mild mosaic virus), VLPs (virus-like particles), OmpC (Outer-membrane protein C), PRRSV (Porcine respiratory and reproductive syndrome virus), HA (hemagglutinin), TIV (trivalent inactivated vaccine).

## Data Availability

Not applicable.
